# Peripheral leukocyte counts and outcomes after intracerebral hemorrhage

**DOI:** 10.1186/1742-2094-8-160

**Published:** 2011-11-16

**Authors:** Shruti Agnihotri, Alexandra Czap, Ilene Staff, Gil Fortunato, Louise D McCullough

**Affiliations:** 1 Department of Neurology, University of Connecticut, 263 Farmington Avenue, Farmington, CT, USA; 2 Department of Research, Hartford Hospital, 80 Seymour Street, Hartford, CT, USA

**Keywords:** Intracerebral Hemorrhage, Outcomes, Inflammation, Leukocyte Count

## Abstract

**Background:**

Intracerebral hemorrhage (ICH) is a devastating disease that carries a 30 day mortality of approximately 45%. Only 20% of survivors return to independent function at 6 months. The role of inflammation in the pathophysiology of ICH is increasingly recognized. Several clinical studies have demonstrated an association between inflammatory markers and outcomes after ICH; however the relationship between serum biomarkers and functional outcomes amongst survivors has not been previously evaluated. Activation of the inflammatory response as measured by change in peripheral leukocyte count was examined and assessment of mortality and functional outcomes after ICH was determined.

**Findings:**

Patients with spontaneous ICH admitted to a tertiary care center between January 2005 and April 2010 were included. The change in leukocyte count was measured as the difference between the maximum leukocyte count in the first 72 hours and the leukocyte count on admission. Mortality was the primary outcome. Secondary outcomes were mortality at 1 year, discharge disposition and the modified Barthel index (MBI) at 3 months compared to pre-admission MBI. 423 cases were included. The in-hospital mortality was 30.4%. The change in leukocyte count predicted worse discharge disposition (OR = 1.258, p = 0.009). The change in leukocyte count was also significantly correlated with a decline in the MBI at 3 months. These relationships remained even after removal of all patients with evidence of infection.

**Conclusions:**

Greater changes in leukocyte count over the first 72 hours after admission predicted both worse short term and long term functional outcomes after ICH.

## Findings

Cerebrovascular disease is the leading cause of morbidity and mortality in the United States. Intracerebral hemorrhage (ICH) is a devastating subtype of stroke, which has worse outcomes than ischemic stroke, is increasing in prevalence, and has a 30 day mortality of 40-50% [1,2]. Only 20% of patients with ICH return to independent function at 6 months [2,3]. Despite advances in management, the mortality of ICH has not changed significantly over time [4]. The role of inflammation in the pathophysiology of ICH is now increasingly recognized. In animal models, a robust inflammatory response is triggered by the entry of blood into the brain parenchyma [5] with a subsequent infiltration of peripheral leukocytes, activation of microglia and release of cytokines [6,7]. Autopsies in both animals [8] and humans [9] with ICH demonstrate leukocytic infiltration usually within first 3 days and inflammatory changes in the penumbra of the hemorrhage. Few clinical studies have demonstrated association between the inflammatory markers and outcomes of ICH in terms of mortality [10,11]. None of these studies evaluated functional or long term outcomes.

We evaluated the relationship between activation of the inflammatory response as measured by change in peripheral leukocyte count, and mortality and functional outcomes after ICH.

This is a retrospective analysis from the existing stroke database at Hartford Hospital, a tertiary care hospital with a primary stroke center in Hartford, CT, USA. The study was reviewed and approved by the Hartford hospital IRB. We included patients with spontaneous ICH from Jan 2005 to April 2010. We excluded subarachnoid hemorrhages, hemorrhages secondary to trauma, malformations, coagulopathy, and tumors as well as patients with leukemia. Baseline characteristics including age, gender, blood pressure, infection within first 72 hours, NIH on admission and ICH score were obtained. The maximum leukocyte counts in the first 72 hours as well as on admission were recorded. The difference between the two values was calculated to obtain the independent variable of change in leukocyte count, which was measured as a continuous variable. The outcomes were measured as in hospital mortality, 12 month mortality, discharge disposition, and the loss on MBI points calculated as a difference between a pre stroke MBI and a MBI at 3 months. Disposition was dichotomized as discharge to worse vs. same or better based on the pre-admit residence. The loss of MBI points was measured as a continuous variable. Data on infection within first 72 hours including that on admission was also collected. Patients with clinical signs and symptoms of infection, including fever, raised peripheral WBC, worsening sensorium, cough, abdominal pain, or diarrhea underwent an infectious work up. The initial work up included urinalysis, chest x-ray, blood cultures, stool for *Clostridium difficile*. If these were abnormal, appropriate cultures were obtained. Those cultures that were identified as bacterial colonization, were excluded from our analysis. Further subgroup analysis after removal of patients with documented infections was also performed. SPSS was used to perform statistical analyses including t-test, linear correlation, and logistic regression. A p value of < 0.05 was considered significant.

There were a total of 423 cases. The in-hospital mortality was 30.4%. The 1 year mortality was 45.2%. The change in leukocyte count information was missing in 68 cases. This was mostly due to elderly patients being made comfort measures only within first 24 hours of admission. Of, the 255 patients alive at 3 months, the MBI data for 3 months was missing in 84 cases. Of the 241 patients alive at 12 months, the MBI data for 12 months was missing in 160 cases. The background demographics and baseline leukocyte information are listed in table 1. Data on infection show that lung and urine infections predominate (Table 2). In univariate analysis (Table 3), the change in leukocyte count was significantly associated with mortality (r = 1.083, p = 0.039) as well as with worse discharge disposition (r = 1.357, p = < 0.001). The change in leukocyte count also correlated with the loss of points on MBI at 3 months compared to a pre-event MBI (r = 0.382, p = < 0.001). In a multivariate logistic regression model (Table 3), the change in leukocyte count was associated with in hospital mortality as well as 12 month mortality with an odds ratio of 1.096 but none were statistically significant. However, it did predict worse discharge disposition with an odds ratio of 1.258 (p = 0.009) after adjustment for various confounders including age, gender, infection, extraventricular drain (EVD) placement, etiology of bleed, ICH score and NIH score on admission. The correlation with the change in MBI score at 3 months remained significant (r = 0.222, p = 0.005) after adjustment for various confounders. The same trend could not be demonstrated for a MBI change at 12 months from the event. A subgroup analysis was performed after excluding all patients with infection within the first 72 hours. The change in leukocyte count over 72 hours remained a significant predictor of a worse discharge disposition (OR = 1.296, p = 0.013)

**Table 1:** Demographics and Leukocyte data

	Frequency (Percentage)
Gender	
Male	204 (48.2)
Female	218 (51.5)

Age	71.67 (13.8)*

ICH volume (in cubic centimeters)	33.90 (41.8)*

NIH on admission	9.96 (7.8)*

Etiology	
Hypertension	315 (74.5)
Amyloid	108 (25.5)

Extraventricular drain	31 (7.3)

First leukocyte count	9.79 (4.17)*

Maximum leukocyte count in the first 72 hours	11.97 (4.56)*

Change in leukocyte count from admission to 72 hours	1.95 (2.71)*

* mean of frequencies (standard deviation)

**Table 2:** Infection data

	Frequency (Percentage)
Infection within 72 hours	55 (13)

Lung infection within 72 hours	31 (7.3)

Urine infection within 72 hours	27 (6.4)

**Table 3:** Change in Leukocyte counts: admission to maximum in 72 hours

	
	**Univariate**			**Multivariate**		

**OUTCOME**	**N**	**Correlation**	**p value**	**N**	**OR/Correlation**	**p value**

Inhospital Mortality	405	1.083	0.039	355	1.096	0.134

Discharge Disposition	404	1.357	0.000	354	1. 258	0.009

1 year mortality	406	1.074	0.055		1.096	0.088

MBI change @ 3 months	179	0.382	0.000	171	0.222	0.005

MBI change @ 12 months	83	0.107	0.334	81	-0.118	0.322

Our results demonstrate that peripheral activation of inflammation predicts outcomes after ICH. Prior clinical studies have examined temperature, admission leukocyte count and C-reactive protein as measures of inflammation and have demonstrated their association with ICH volume and mortality [10,11]. Previous studies have documented that higher peripheral leukocyte counts are associated with early neurologic deterioration but were not independently associated with functional outcomes as measured by modified rankin scale at 30 days [12]. One recent study found that although a higher white blood cell count was associated with increased mortality in ICH patients, but this did not increase the risk of death independently of other indicators of ICH severity [10]. In contrast to our work, no association was seen between median leukocyte count and functional outcome at 30 days [10]. This may be due to the two-fold larger sample size and the use of change in WBC over 72 hours rather than use of the median WBC count in this study. The robustness of our data is evident in the fact that in the subgroup analysis where infections are excluded, the association between change in leukocyte count and the discharge disposition remains significant.

Peripheral leukocytes are a marker of the response of the immune system and reflect the activation of the inflammatory cascade following a spontaneous ICH [13]. This is an easily and widely available biomarker. The change in the leukocyte count over 72 hours may better reflect the amount of inflammatory response mounted in response to the ICH compared to a single value. The use of this variable is advantageous as it reduces the impact of premorbid conditions that affect the leukocyte count, and may more accurately reflect the amount of neuroinflammation as it remained predictive even when adjusted for infections on admission as well as those that developed within 72 hours of presentation.

In our study, the peripheral leukocyte count independently predicts poor functional outcomes in terms of discharge disposition and the loss of points on the MBI at 3 months. This suggests that activation of peripheral immune system may enhance injury after ICH, consistent with emerging experimental work [13]. We could not establish an association with mortality and it is most likely due to loss of leukocyte count data on patients who were made hospice early on in the care. Similarly, we could not establish a trend for MBI at 12 months largely due to missing information which is a limitation in this study. However, the change in leukocyte count was predictive of outcome at 3 months, one of the longest outcome assessments correlated with a biomarker to date in ICH patients.

There are several limitations of this study which include the retrospective design, single center population and missing data on certain variables, especially at 12 months, largely due to the high mortality from this disease. However, this is one of the largest samples to date, and clearly demonstrated the association between the peripheral immune response and poor functional outcomes after ICH independent of infection or hemorrhage size. Treatments for ICH have focused on blood pressure management and reducing ICH volume [14]. None of these treatments have made a significant impact on patient outcome. Mortality and morbidity have not changed in several decades, despite improvements in ICU care. This study provides further evidence for the potential of targeting neuroinflammation as a treatment modality to improve outcomes amongst ICH survivors.

## Competing interests

The authors declare that they have no competing interests.

## Authors' contributions

LM: Conceived the study, revised the manuscript. SA: Participated in study design and co-ordination, drafted the manuscript. AC: Performed data-collection and chart reviews. IS: Performed statistical analysis. GF: Performed data extraction and database maintenance. All authors have read and approved the final manuscript.
